# Lack of Effect of Methylene Blue in the SOD1 G93A Mouse Model of Amyotrophic Lateral Sclerosis

**DOI:** 10.1371/journal.pone.0023141

**Published:** 2011-10-06

**Authors:** Rosamond Lougheed, John Turnbull

**Affiliations:** Department of Medicine, McMaster University, Hamilton, Ontario, Canada; Johns Hopkins, United States of America

## Abstract

**Background:**

Methylene blue (MB) is a drug with a long history and good safety profile, and with recently-described features desirable in a treatment for ALS.

**Methodology/Principal Findings:**

We tested oral MB in inbred high-copy number SOD1 G93A mice, at 25 mg/kg/day beginning at 45 days of age. We measured disease onset, progression, and survival. There was no difference in disease onset between MB-treated mice and controls, although subgroup analysis showed a modest but statistically significant delay in disease onset in MB-treated female mice only (control 122±10.2 versus MB 129±10.0 days). MB-treated mice of both sexes spent more time in less severe stages of disease, and less time in later, more severe stages of disease. There was a non-significant trend to longer survival in MB-treated animals (control males reached endpoint at 161±14.1 days, versus 166±10.0 days for MB-treated animals, and control females reached endpoint at 171±6.2 days versus 173±13.4 days for MB-treated animals).

**Conclusions/Significance:**

In spite of a strong theoretical rationale, MB had no significant effects on onset or survival in the inbred SOD1 G93A mouse model of ALS.

## Introduction

ALS is a fatal disease characterized by progressive weakness of voluntary muscle. Most cases of ALS are sporadic and without known cause. Some cases are familial (FALS) and of these, a minority are associated with known mutation, in genes encoding SOD1 [1], Alsin [2], Dynactin 1 [3], VAPB [4], angiogenin [5], TDP43 [6], [7], or FUS [8], [9] Mutation in genes more commonly associated with other diseases can rarely present as ALS (eg. Spastin [10]) as can viral infection (eg. HIV [11] and HTLV [12]). It is presently unclear how multiple known and unknown triggers can lead to the same disease phenotype. However, several pathological mechanisms may be in common, including impaired axonal transport and reduced trophic support, excitotoxicity, oxidative stress, mitochondrial dysfunction, inflammation, accelerated ageing, and terminally, apoptosis. No intervention in ALS targeting any of these processes singly has proven successful in substantially mitigating the disease process. It is possible that these processes are secondary to a single more fundamental disease process not yet discovered. Presently, however, ALS may best be treated with therapies that target multiple points in the disease cascade.

Methylene Blue is an old drug with several properties of interest in a potential ALS therapy. MB readily alternates between reduced and oxidized forms (eg. Wiklund [13]), and it has been successfully used as a surrogate electron transporter in patients with impairment of the mitochondrial electron transport chain (ETC). Thus, MB has a long use in cyanide poisoning, where Complex IV of the ETC is blocked. Functional defects in ETC complexes I and IV have been identified in ALS patients [14], and MB could overcome these deficits.

MB pretreatment reduces brain damage and death following experimental cardiac arrest in pigs [13]. This effect was attributed to a reduction in the action of nitric oxide (NO), and decreased glial activation and lipid peroxidation. MB inhibits nitric oxide synthase [15]. The synthesis of NO is increased in all studied SOD1 FALS mutations, an effect not seen with over-expression of wild-type SOD1 [16], and cellular damage in ALS has been linked to increased levels of NO (eg. Beal [17], Ferrante [18]).

Similarly, MB protects cultured fibroblasts against H_2_O_2_ challenge, an effect attributed in part to the induction of phase-2 antioxidant defense enzymes including NADPH dehydrogenase quinone 1 and the selenoprotein thioredoxin reductase 1 (Trx1) [19]. Selenoproteins act as anti-oxidants, in signaling pathways, and in the maintenance of intracellular REDOX balance [20], [21]. Markers of oxidative stress and lipid and protein peroxidation are all elevated in ALS [22], [23], and could be mitigated by MB.

MB reduces pathological microtubule-associated protein (MAP) tau aggregation [24], and may thereby improve axonal function, a matter of considerable interest in Alzheimer's Disease (AD). In a recent Phase II study of 321 patients with AD, MB slowed disease progression by 81% [25]. Intraneuronal hyperphosphorylated tau deposits are seen in ALS [26], [27] and could contribute to the impaired axonal transport and axonal dysfunction that characterize both sALS and FALS [28], [29]. Impairments in retrograde axonal transport could result in a deficiency of trophic support to the motor neuron.

Last, MB is one of the few compounds capable of significantly prolonging lifespan in normal mice and rats, and it does so in a dose related fashion [30]. The mechanism of this effect is unclear, but of potential relevance to ALS since it has long been suggested that ALS is due to an accelerated senescence of motor neurons (eg. McComas [31]).

Even at the highest dose of MB (25 mg/kg) administered chronically in the lifespan study in mice and in rats, only minor toxicity was seen. At usual doses, MB produces few side effects in humans [19]. Since MB favorably affects several of the pathways affected in ALS and since it is readily bioavailable to the central nervous system following oral administration, and since the drug is already approved for human use, we tested MB in a mouse model of ALS. Because of concerns of variability in outcome measures using the standard SOD1 G93A hybrid mouse model raised by Scott *et al* [32], we used a more recently developed inbred (congenic) SOD1 G93A strain for these experiments. We measured the age at disease onset, disease progression (using a customized 7-point clinical scale), and age at surrogate death.

## Results

Daily oral administration of MB was started at 45 days of age and continued until death. 26/27 treated animals and 19/20 control animals completed the study according to protocol. One control group animal and one drug group animal were sacrificed early at the request of the University veterinarian for reasons not related to weakness (skin conditions) and censored from the study as non-ALS deaths. There were no discernable side effects to MB treatment.

**Figure 1:**
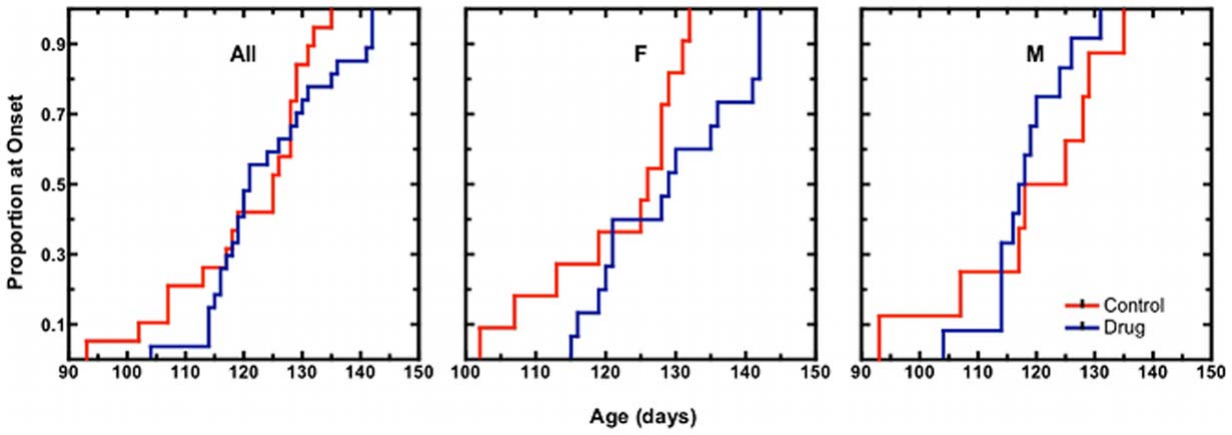
Kaplan-Meier plots for onset of neurological signs in congenic SOD1 G93A mice. Clinical onset of disease is taken as the age at which each mouse attained a neurological severity score of 1 (please refer to Materials and Methods for a detailed description of assignment of neurological scores). The proportion of mice reaching onset over time is shown for all mice (All), females only (F), and in males only (M). Methylene blue treated (MB) animals received daily 25 mg/kg doses of methylene blue dissolved in Jello®. Vehicle control (Control) animals received unaltered Jello® cubes.

The Kaplan Meier curve for age at disease onset (score 1 on our disease activity scale) is shown in Figure 1, for all mice, and then separately by sex. Control mice first showed neurological signs at 121±11.5 days (mean± SD; n = 19). This falls between onset values reported by Wooley *et al.* [33] (at 142+/−10 days) and those of Hayworth and Gonzalez-Lima [34] (at 100+/−2 days and 91+/−2 days for females and males, respectively) in the same mouse strain. Animals receiving MB showed first signs of disease slightly later, at 124±10.3 days (n = 27). This difference was not significant (log rank p>0.05). However, when stratified by sex, MB treated female mice showed signs of disease at 129±10.0 days (n = 15), compared with 122±10.2 days (n = 11) in female controls. This difference was significant (log rank p<0.05). There was no difference in male mice (118±6.9 days MB, n = 12 versus 119±13.6 days, n = 8 control).

**Figure 2:**
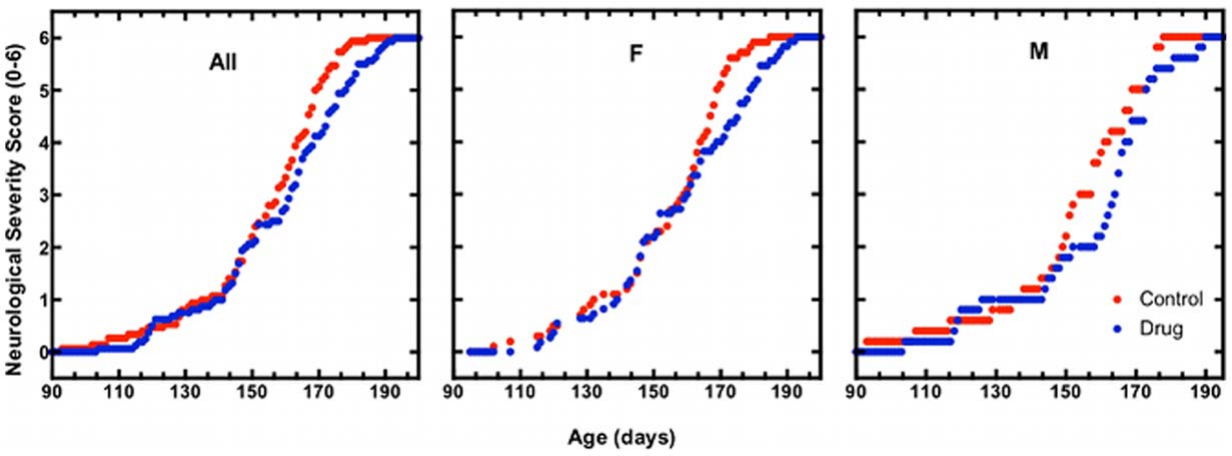
Daily average neurological severity score in congenic SOD1 G93A mice. Group average neurological severity scores across the age from the start of the study to the age at endpoint in all mice (All; *N_Control_* = 15, *N_MB_* = 16), females only (F; *N_Control_* = 10, *N_MB_ = 10*), and males only (M; *N_Control_* = 5, *N_MB_ = 6*). Methylene blue treated (MB) animals received daily 25 mg/kg doses of methylene blue dissolved in Jello®. Vehicle control (Control) animals received unaltered Jello® cubes.

The average daily disease activity score for MB and control mice as a function of age is shown in Figure 2. At most ages, MB-treated animals had less average disease severity than control mice. We quantified this by tabulating the average length of time spent at each score (Table 1). MB treated animals spent more time in the less severe stages and less time in more severe stages than untreated animals, although the effect was significant only where indicated by an asterisk.

**Table 1:** Neurological Severity Score in Congenic SOD1 G93A mice: Categorical Analysis of Average Number of Days at Score by Treatment Group.^1^^,^ 2

Subjects	Parameter	Treatment	Score 0	Score 1	Score 2	Score 3	Score 4	Score 5	Score 6
All	Mean Frequency	Control	118.6	25.8	11.67	6.26	3.33	2.6	1.0
		Drug	121.4	24.3	14.88	7.19	1.75	1.56	1.0
	P value		0.239	0.388	0.014*	0.323	0.005*	0.458	1.0
Female	Mean Frequency	Control	120.3	23.9	12.3	6.2	3.3	2.4	1.0
		Drug	117.3	21.64	13.8	7.91	1.73	2.27	1.0
	P value		0.525	0.277	0.336	0.141	0.022*	0.849	1.0
Male	Mean Frequency	Control	115.2	29.6	10.4	6.4	3.4	3.0	1.0
		Drug	116.4	30.0	17.2	5.6	1.8	2.0	1.0
	P value		0.860	0.907	0.036*	0.605	0.114	0.316	1.0

1 Testing Terms: Scores for Control (unaltered Jello®) and Drug (25 mg/kg methylene blue daily) are given as mean number of days spent within each severity category. P value lists the probability of obtaining, by chance alone, a value greater than the one computed if the score frequencies are the same for both treatment groups at group size minus one degree of freedom (test term description taken from the JMP® 7.0.1 Help file).

2 Refer to Material and Methods for a detailed description of assignment of neurological scores. Neurological disease severity increases from 0–6. Score 0 is asymptomatic. Score 6 is surrogate endpoint.

**Figure 3:**
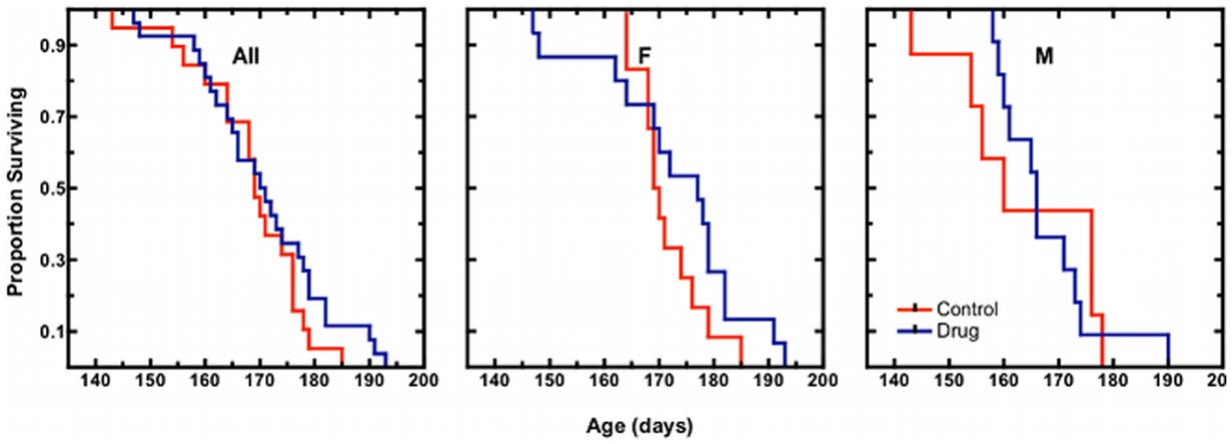
Kaplan-Meier plots for survival time in congenic SOD1 G93A mice. Mice are considered moribund and are given a neurological severity score of 6 at the age at when they can no longer right themselves after 30 seconds of being placed on either side (please refer to Materials and Methods for a detailed description of assignment of neurological scores). The proportion of mice reaching endpoint over time is shown for all mice (All), females only (F), and males only (M). Methylene blue treated (MB) animals received daily 25 mg/kg doses of methylene blue dissolved in Jello®. Vehicle control (Control) animals received unaltered Jello® cubes.

MB mice reached endstage (our score 6) at 170±12.3 days (n = 27; Figure 3) slightly later than control mice, at 167±11.1 days (n = 20). This difference was not significant (log rank p*>*0.05). When stratified by sex, both males and females treated with MB survived marginally longer than controls, although the difference was not significant for either group. (MB females reached endpoint at 173±13.4 days (n = 15) and control females at 171±6.2 days (n = 12). MB males reached endpoint at 166±10.0 days (n = 12) and control males at 161±14.1 days (n = 8)).

## Discussion

MB has multiple properties that should make it an interesting candidate treatment for ALS. As set out above, MB could overcome the mitochondrial ETC defect seen in ALS, reduce glial activation and inflammatory responses, reduce oxidative damage, and improve axonal dysfunction, and even in normal animals, MB significantly prolongs lifespan.Thus, MB should favourably affect many of the pathological processes thought to be operative in ALS, and we held some optimism that it would be successful in an ALS disease model.

Indeed, SOD1 G93A mice treated with MB did show delayed disease onset, spend more time in less severe stages and less time in more severe stages of disease, and die at a later age than control mice. However, while the effects are minor, often not reaching statistical significance, and certainly not of the magnitude that we had hoped for. The lack of success can be explained in two ways. It may be that MB is of minimal utility in ALS, or, it may be that the model used is flawed.

No single underlying pathogenic mechanism has been forthcoming to explain the multiple pathogenic abnormalities seen in ALS (impaired axonal transport and reduced trophic support, excitotoxicity, oxidative stress, mitochondrial dysfunction, inflammation, accelerated ageing, and terminally, apoptosis), and unfortunately, all attempts targeting these processes singly have been disappointing in humans and, even when statistically significant in mouse models, the effect is always modest. We had hoped that MB, targeting several of these processes, might be more successful, but this was not the case. This reinforces the possibility that satisfactory therapy must await the discovery of more fundamental proximal causes of pathogenesis. One possibility presently unfolding is disordered RNA processing (eg Kolb [35]), which might underlie many diseases of the motor neuron, and which might directly or indirectly disrupt many downstream processes.

Conversely, it is possible that some of the difficulty lies with our choice of parameters or the SOD1 mouse model itself. We administered MB at a dose of 25 mg/kg/d. We chose this dose because it was the highest dose given chronically in the above referenced longevity study [30], and the dose that produced the greatest beneficial effect. It is possible that the optimal dose in a longevity study might differ from an optimal dose in SOD1 mice, and that a different (larger) dose might have produced greater benefit. However, the authors of the longevity did consider doses higher than 25 mg/kg/d, but limiting toxicity was seen in plreliminary studies, thereby precluding the use of higher doses in chronic administration.

Also, in the longevity study, MB was administered daily by gavage. On the advice of our institutional review board and University veterinarian, we substituted daily oral administration of MB in flavored gelatin (Jello®), as less stressful to the animals. The animals enthusiastically consumed the Jello® containing the MB, and we have no reason to suspect that the Jello® would alter the bioavailability of MB from the gut. Indeed, MB is administered to humans in gelatin capsules [36], and several previous studies have show that the oral bioavailability of MB is high. Pharmacokinetic studies in mice and rats show it is well absorbed from the gastrointestinal tract, readily crosses the blood-brain barrier, and concentrates in the brain [37]–[39]. (One hour after duodenal administration of MB, the brain concentration exceeds the whole blood concentration by a factor of ten [39]).

However, assuming that there are no major difficulties with either dose or administration, it is possible that the difficulty lies with the SOD1 G93A mouse model. This model is subject to large endpoint variability, as studied by Scott et al [32]. They concluded that most of the G93A mouse studies to date have been underpowered, with the reported success of many agents due to the play of chance and presumably a bias towards reporting positive results. When they retested compounds reported positive in previous studies, with greater numbers of animals, matching of sex and litter, and with control of the transgene copy number, none of the studies were positive. If true, two solutions to this are thus possible: one can increase the rigour with which experiments are undertaken as above, or use a model with less inherent variability.

We did both. We aimed for a high number of animals per treatment group, and matched them for sex and litter. Also, we used an inbred G93A SOD1 mouse model, which should have less background variability in outcome measures than the hybrid strain. Indeed, this does seem to be the case. The average standard deviation in survival reported in the control arms using the hybrid G93A mouse averages ±12 days [40], or perhaps more meaningfully, 8.8% of the reported lifespan. In our hands, the standard deviation of the inbred (congenic) controls was ±10.6 days, or 5.9% of the reported lifespan. This reduction in variability should improve reliability and reduce the number of animals needed for a given power. (Indeed, using these figures, one can calculate that for customary values (alpha  =  0.05 and beta  =  0.2), if 24 mice are recommended for a given effect size in the hybrid mouse model, then 11 per group would be needed in the inbred strain, a significant reduction.)

However, we found these animals difficult to use. The inbred B6 dams tended to have poor parenting skills and cannibalized pups with even minor environmental stress. We lost over 100 pups in this way, before resorting to surrogate timed-pregnant ICR dams, which greatly added to the expense of the study. The same power might be achieved more simply by assuring an adequate number of the usual SOD1 animals and controlling for copy number, litter and sex (as suggested by Scott *et al.* [32]), with equal or less expense.

It is also possible that the SOD1 G93A mouse suffers more fundamental difficulties than endpoint variability. Multiple agents have shown some (albeit modest) benefit in the hybrid G93A mouse model, yet none have translated to therapeutic success in humans, and it seems unlikely that chance alone and a bias to positive reporting could explain this. Another explanation is that the SOD1 mouse may be a good model for SOD1 FALS, but not other familial ALS or sporadic disease. The development of TDP43 mice, which might better reflect most human ALS, might afford a truer disease model than the SOD1 mouse, and it may be useful to revisit promising agents minimally successful in SOD1 studies (such as MB), in TDP43 mice.

## Materials and Methods

### Ethics Statement

All animal experiments and procedures were carried out with the approval of McMaster University's Animal Research Ethics Board, as per the Animal Utilization Protocol 09-11-43.

High-copy SOD1 G93A congenic breeding pairs were obtained from the Jackson Laboratory (B6.Cg-Tg(SOD1*G93A)1 Gur/J, stock number, 004435). We maintained the colony by crossing hemizygous male carriers with inbred wild-type C57BL/6 dams. These dams tended to cannibalize their pups with even minor environmental stress, and in consequence we transferred litters shortly after birth to timed pregnant surrogate ICR dams (Institute for Cancer Research dams; Charles River, Wilmington, Massachusetts; strain name: Crl:CD1 (ICR), stock number: 022). Mice were individually housed in the McMaster University Central Animal Facility in a 12-h light/dark cycle. Food and water were available *ad libitum* throughout the study.

Mice were randomly assigned to receive either MB or no MB (control) at 45 days of age. Allocation was balanced by litter and sex. MB (Sigma-Aldrich M44907) was dissolved in 3 ml warm freshly prepared raspberry Jello® solution (at the recommended Jello® concentration), while control Jello® was prepared without MB. Liquid Jello® with or without MB was poured into 3 mL molds, and the solidified cubes were then placed on the bottom of each animal's cage on a daily basis. Fresh Jello® was made three times a week, with each treated animal receiving approximately 25 mg/kg/day MB. Cages were checked each morning to ensure that the Jello® cubes had been entirely consumed. (They always were).

All mice were examined daily from 45 days of age to endpoint. After symptom onset animals were monitored twice daily. Body weight measurements were monitored periodically prior to onset of overt symptoms, and weekly afterwards. We used a single 7 point descriptive scale to assess disease progression. To reduce subjective variability, all assessments were done by a single examiner (RL).

- 0 Asymptomatic.
- 1 Trembling/shaking of hindlimbs during suspension of mouse by its tail, and full/partial collapse of leg extension towards lateral midline. This is the age of onset usually reported [41].
- 2 Impairment of gait with respect to stride length and speed when mouse is placed on a flat surface.
- 3 Mouse can be placed on its side but rights itself immediately.
- 4 When placed in its side, mouse takes between 2-15 seconds to right.
- 5 When placed in its side, mouse takes between 16-30 seconds to right.
- 6 End-stage disease. The mouse is unable to right itself within 30 seconds after being placed in its side. At this point the animals are euthanized by cervical dislocation.

Kaplan-Meier survival fit analysis with log-rank statistics were used to analyze the time-to-event measures (onset and survival). Categorical analysis was used to analyze the daily ordinal neurological severity score. Differences in score frequencies were analyzed by Chi Square. (The last scores of euthanized animals were carried forward.) All statistical tests were carried out using GraphPad Prism 4.0®, GraphPad Software, Inc., San Diego, CA, USA and JMP® 8.0, SAS Institute, Inc., SAS Campus Drive, Cary, NC 27513, USA. Two-tailed p values less than 0.05 were considered to be statistically significant.

## References

[1] Rosen D, Siddique T, Patteron D, Figlewicz D, Sapp P (1993). Mutations in Cu/Zn superoxide dismutase gene are associated with familial amyotrophic lateral sclerosis.. Nature.

[2] Yang Y, Hentati A, Deng H, Dabbagh O, Sasaki T (2001). The gene encoding alsin, a protein with three guanine–nucleotide exchange factor domains, is mutated in a form of recessive amyotrophic lateral sclerosis.. Nat Genet.

[3] Munch C, Sedlmeier R, Meyer T, Homberg V, Sperfeld A (2004). Point mutations of the p150 subunit of dynactin (DCTN1) gene in ALS.. Neurology.

[4] Nishimura A, Mitne-Neto M, Silva H, Richieri-Costa A, Middleton S (2004). A mutation in the vesicle trafficking protein VAPB causes late-onset spinal muscular atrophy and amyotrophic lateral sclerosis.. Am J Hum Genet.

[5] Greenway M, Andersen P, Russ C, Ennis S, Cashman S (2006). ANG mutations segregate with familial and ‘sporadic’ amyotrophic lateral sclerosis.. Nat Genet.

[6] Neumann M, Sampathu D, Kwong L, Truax A, Micsenyi M (2006). Ubiquitinated TDP-43 in frontotemporal lobar degeneration and amyotrophic lateral sclerosis.. Science.

[7] Arai T, Hasegawa M, Akiyama H, Ikeda K, Nonaka T (2006). TDP-43 is a component of ubiquitin-positive tau-negative inclusions in frontotemporal lobar degeneration and amyotrophic lateral sclerosis.. Biochem Bioph Res Co.

[8] Vance C, Rogelj B, Hortobagyi T, De Vos K, Nishimura A (2009). Mutations in FUS, an RNA Processing Protein, Cause Familial Amyotrophic Lateral Sclerosis Type 6.. Science.

[9] Kwiatkowski T, Bosco D, Leclerc A, Tamrazian E, Vanderburg C (2009). Mutations in the FUS/TLS gene on chromosome 16 cause familial amyotrophic lateral sclerosis.. Science.

[10] Meyer T, Schwan A, Dullinger J, Brocke J, Hoffman K (2005). Early-onset ALS with long-term survival associated with spastin gene mutation.. Neurology.

[11] McGowan D, Scelsa S, Waldron M (2001). An ALS-like syndrome with new HIV infection and complete response to antiretroviral therapy.. Neurology.

[12] Silva M, Leite C, Alamy A, Chimelli L, Andrada-Serpa M (2005). ALS syndrome in HTLV-I infection.. Neurology.

[13] Wiklund L, Basu S, Micescu A, Wiklund P, Ronquist G (2007). Neuro- and cardioprotective effects of blockade of nitric oxide action by administration of methylene blue.. Ann NY Acad Sci.

[14] Wiedemann F, Winkler K, Kuznetsov A, Bartels C, Vielhaber S (1998). Impairment of mitochondrial function in skeletal muscles of patients with amyotrophic lateral sclerosis.. J Neurol Sci.

[15] Mayer B, Brunner F, Schmidt K (1993). Inhibition of nitric oxide synthesis by methylene blue.. Biochem Pharmacol.

[16] Almer G, Vukosavic S, Romero N, Przedborski S (1999). Inducible nitric oxide synthase up-regulation in a transgenic mouse model of familial Amyotrophic Lateral Sclerosis.. J Neurochem.

[17] Beal M, Ferrante R, Browne S, Matthews R, Kowall N (1997). Increased 3-nitrotyrosine in both sporadic and familial amyotrophic lateral sclerosis.. Ann Neurol.

[18] Ferrante R, Shinobu L, Schulz J, Matthews R, Thomas C (1997). Increased 3-nitrotyrosine and oxidative damage in mice with a human copper/zinc superoxide dismutase mutation.. Ann Neurol.

[19] Atamna H, Nguyen A, Schultz C, Boyle K, Newberry J (2008). Methylene blue delays cellular senescence and enhances key mitochondrial biochemical pathways.. FASEB.

[20] Jilani K, Panee J, He Q, Berry M, Li P (2007). Overexpression of Selenoprotein H Reduces HT22 Neuronal Cell Death after UVB Irradiation by Preventing Superoxide Formation.. Int J Biol Sci.

[21] Papp L, Holmgren A, Khanna K (2010). Selenium and selenoproteins in health and Disease.. Antioxid Redox Sign.

[22] Andrus P, Fleck T, Gurney M, Hall E (1998). Protein oxidative damage in a transgenic mouse model of familial amyotrophic lateral sclerosis.. J Neurochem.

[23] Barber S, Mead R, Shaw P (2006). Oxidative stress in ALS: a mechanism of neurodegeneration and a therapeutic target.. Biochim Biophys Acta.

[24] Harrington C, Rickard J, Horsley D, Harrington K, Hindley K (2008). Methylethioninium chloride (MTC) acts as a tau aggregation inhibitor (TAI) in a cellular model and reverses tau pathology in transgenic mouse models of Alzheimer's disease.. Alzheimer's Dement.

[25] Gura T (2008). Hope in Alzheimer's fight emerges from unexpected place.. Nat Med.

[26] Yang W, Sopper M, Leystra-Lutz C, Strong M (2003). Microtubule associated tau protein positive neuronal and glial inclusions in amyotrophic lateral sclerosis.. Neurology.

[27] Strong M, Yang W, Strong W, Leystra-Lantz C, Jaffe H (2006). Tau protein hyperphosphorylation in sporadic ALS with cognitive impairment.. Neurology.

[28] Sasaki S, Iwata M (1996). Impairment in fast axonal transport in the proximal axons of anterior horn neurons in amyotrophic lateral sclerosis.. Neurology.

[29] Fischer L, Culver D, Tennant P, Davis A, Wang M (2004). Amyotrophic lateral sclerosis is a distal axonopathy: evidence in mice and man.. Exp Neurol.

[30] National Toxicology Program (2008). NTP toxicology and carcinogenesis studies of methylene blue trihydrate (CAS No. 7220-79-3) in F344/N rats and B6C3F1 mice (Gavage Studies).. Natl Toxicol Prog Tech Rep Ser.

[31] McComas A, Upton A, Sica R (1973). Motoneuron disease and ageing.. Lancet.

[32] Scott S, Kranz J, Cole J, Lincecum J, Thompson K (2008). Design, power, and interpretation of studies in the standard murine model of ALS.. Amyotroph Lateral Sc..

[33] Wooley C, Sher R, Kale A, Frankel W, Cox G (2005). Gait analysis detects early changes in transgenic SOD1(G93A) mice.. Muscle Nerve.

[34] Hayworth C, Gonzalez-Lima F (2009). Pre-symptomatic detection of chronic motor deficits and genotype prediction in congenic B6.SOD1G93A ALS mouse model.. Neuroscience.

[35] Kolb S, Sutton S, Schoenberg D (2010). RNA processing defects associated with diseases of the motor neuron.. Muscle Nerve.

[36] Oz M, Lorke DE, Hasan M, Petroianu GA (2010). Cellular and molecular actions of methylene blue in the nervous system.. Med Res Rev.

[37] O'Leary J, Qingyou L, Marinec P, Blair L, Congdon E (2010). Phenothiazine-mediated rescue of cognition in tau transgenic mice requires neuroprotection and reduced soluble tau burden.. Mol Neurodegener.

[38] Walter-Sack I, Rengelshausen J, Oberwittler H, Burhenne J, Mueller O (2009). High absolute bioavailability of methylene blue given as an aqueous oral formulation.. Eur J Clin Pharmacol.

[39] Peter C, Hongwan D, Kupfer A, Lauterburg B (2000). Pharmacokinetics and organ distribution of intravenous and oral methylene blue.. Eur J Clin Pharmacol.

[40] Gurney M (1997). The use of transgenic mouse models of amyotrophic lateral sclerosis in preclinical drug studies.. J Neurol Sci.

[41] Gurney M, Pu H, Chiu A, Dal Canto M, Polchow C (1994). Motor neuron degeneration in mice that express a human Cu,Zn superoxide dismutase mutation.. Science.

